# The Origins of Concentric Demyelination: Self-Organization in the Human Brain

**DOI:** 10.1371/journal.pone.0000150

**Published:** 2007-01-17

**Authors:** Roman H. Khonsari, Vincent Calvez

**Affiliations:** 1 Service de Chirurgie Maxillofaciale, Centre Hospitalier Universitaire de Nantes, Nantes, France; 2 Laboratoire de Neuropathologie Raymond-Escourolle, Hôpital de la Pitié-Salpêtrière, Paris, France; 3 Département de Mathématiques et Applications, École Normale Supérieure, Paris, France; University of Sheffield, United Kingdom

## Abstract

Baló's concentric sclerosis is a rare atypical form of multiple sclerosis characterized by striking concentric demyelination patterns. We propose a robust mathematical model for Baló's sclerosis, sharing common molecular and cellular mechanisms with multiple sclerosis. A reconsideration of the analogies between Baló's sclerosis and the Liesegang periodic precipitation phenomenon led us to propose a chemotactic cellular model for this disease. Rings of demyelination appear as a result of self-organization processes, and closely mimic Baló lesions. According to our results, homogeneous and concentric demyelinations may be two different macroscopic outcomes of a single fundamental immune disorder. Furthermore, in chemotactic models, cellular aggressivity appears to play a central role in pattern formation.

## Introduction

Baló's concentric sclerosis has been a neuropathologic enigma since its description in 1927 [1]. Many hypotheses have been formulated to explain its striking, reproducible patterns. Early analogies with the phenomenon of Liesegang ring formation were proposed [2]. Our approach reconsiders these analogies and proposes a non-linear mechanism of self-organization involving a minimal number of assumptions on the course of the disease.

### Neuropathology of Baló's concentric sclerosis

The typical neuropathological lesion of multiple sclerosis (MS) is a spherical or cylindrical perivascular demyelinated zone in the hemispheric or medullar white matter. Myelin sheath depletion generally occurs within this sharply delimited area without major axonal injury. The final effectors of demyelination are the microglia, but the initial trigger that leads to abnormal microglial activation is unknown [3]. The demyelination pattern is seldom heterogeneous.

**Figure 1:**
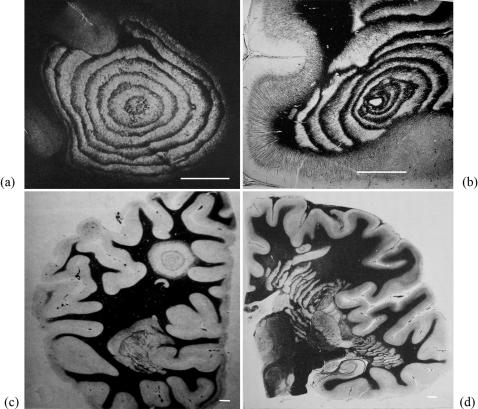
Typical aspects of Baló's concentric sclerosis. (a) Original case of Baló; several anastomoses are located in the lower half of the lesion (from Baló (1928) Arch Neurol Psychiatr 19:242–264). (b) Lesion centered by a veinule showing ring fragmentation in a constrained area (from Hallervorden et al. 1933). (c) Lesion reproduced from Castaigne et al. (1984) Rev Neurol 140:479–487. (d) Progress of the pathologic process from a center located in a constrained area, showing formation of bands (from Behr (1950) Dtsch Z Nervenheilk 164:480–489). Loyez staining (myelin in black, destroyed areas in white); scale bars: 1 cm.

Concentric demyelination, also known as Baló's concentric sclerosis [1], was initially described in acute, rapidly fatal, clinical MS forms. Due to the improvement of MRI resolution, concentric demyelination is now observed in chronic forms of MS, occurring along with classical homogeneous lesions [4]. Baló's lesions are perivascular bundles of concentric demyelinated zones in which areas where myelin is preserved regularly alternate with zones where myelin is destroyed (figure 1). The external border of the lesion bands is sharp, but their internal limit with the normal white matter is not well defined. An MRI follow-up study of Baló patients has shown that the demyelinated layers form progressively in a centrifugal way [5]. More recent results nevertheless indicate that the process of active demyelination takes place synchronously rather than successively, with a progressive evolution of the lesions towards homogeneous MS plaques [6]. Furthermore, MR spectroscopic studies have confirmed the analogies between Baló's sclerosis and classical MS [7]. Baló's sclerosis may thus be a borderline form – or even an intermediate form – of typical MS.

MS is now classified in four subtypes according to the supposed pathologic pathway leading to the microglial activation [8]. Baló's sclerosis belongs to subtype III, in which the oligodendrocytes are the disease's primary targets. Quantitative studies have in fact confirmed important oligodendrocyte depletion in Baló lesions, predominantly in the demyelinated bands [9]. In MS type III lesions, the oligodendrocytes are the victims of a mitochondrial dysfunction creating local ischemic conditions [10]. Primary oligodendrocyte injury has also recently been proposed as the initial pathogenic event in MS lesion development, and early MS lesions some times show concentric patterns of demyelination [11]. Interestingly, concentric lesions in subjects who do not suffer from Baló's sclerosis are described in either ischemia, such as stroke [12] and cyanide poisoning (in cats and rabbits, [13]), or in cases where a specific oligodendrocyte destruction occurs, such as attacks by JC [14] and HHV6 [15] viruses. Concentric sclerosis and very early MS may thus both be the consequence of oxydative stress of oligodendrocytes.

The origins of concentric demyelination have puzzled generations of neuropathologists, and many interesting etiologic hypotheses have been formulated. Courville [12] has argued that the lesions may be the direct result of microthrombosis in the brain capillary network. His vascular theory is probably not valid as no concentric pattern in the blood vessel distribution exists in the human brain. Preserved myelin bands were then attributed to remyelination processes [16], but neuropathological arguments have proven this hypothesis incorrect [17]. Recently, the periodic preservation of myelin in a radially expanding myelinoclastic process has been attributed to a preconditioning phenomenon [18]. According to these authors, a mitochondrial dysfunction triggers local protection mechanisms with a narrow efficiency range around the actively demyelinating zone. Proteins involved in ischemic preconditioning have in fact been found at the periphery of expanding Baló lesions. Wiendl et al. [19] have reported remarkable homogeneous diffusion abnormalities in MRI scans before any concentric contrast enhancement, in areas of acute lesion development. These abnormalities may correspond to an early cytotoxic oedema, and contradict the hypothesis of a strictly radially progressing pathological process. The diffusion abnormality is a very early event and it is not reported by all authors [20].

The best model for testing the pathogenic hypothesis on Baló's sclerosis would be the experimental allergic encephalomyelitis of the common marmoset, where concentric lesions with high oligodendrocyte depletion and massive macrophage recruitment are reported [21].

### Baló's concentric sclerosis and Liesegang rings

The interesting analogy between Baló's sclerosis and the Liesegang ring formation phenomenon has already been thoroughly studied [2], [22]. Liesegang ring formation is a periodic precipitation process initially described in gels. Three chemical species are involved in the following order:
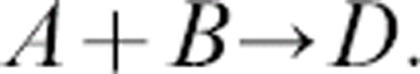
Initially, *B* (for example *AgNO*_3_) is uniformly distributed in the gel and *A* (for example *HCl*) propagates within a diffusion front. As the reaction goes on, consecutive bands of precipitate *D* (*AgCl* in our example) form. Many theories have been proposed to explain this process. One of the earliest and most successful is Ostwald's *supersaturation theory* [23], which is based on a spatially periodic nucleation phenomenon. Precipitation occurs whenever the concentration of compound *AB* exceeds a *supersaturation threshold* q* and takes place as long as [*AB*]>*q*, *q* being the *saturation threshold*. Ostwald's theory postulates that each precipitated band of *D* depletes the surrounding gel of *B* by acting as a sink and thus creates a zone spared by the precipitation front by lowering [*AB*] under q* [24].

The position of the *n^th^* band, *x_n_*, and the time *t_n_* elapsed before the precipitation of the *n^th^* band obey generic laws. The *time law* states that *x_n_* is proportional to 
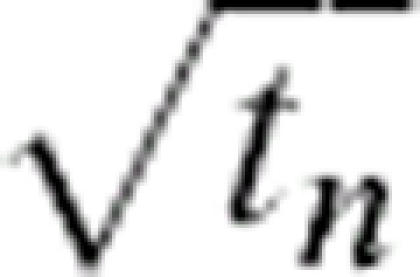
 (diffusion scale). The *spacing law* indicates that the ratio between the position of two successive bands converges to a finite value 1+*p*, where *p*>0. The width of the *n^th^* band increases with *n* and obeys the *width law* *x*_*n*_^*α*^ where α is close to one. The precipitation pattern also depends on geometry – bands are formed in test tubes and rings appear in Petri dishes [25].

Many experimental facts cannot be explained by Ostwald's supersaturation theory, such as the *secondary banding* – one band breaks into narrower bands – and the *inversed spacing* – the spacing between the band decreases [25]. Other irregular patterns are observed in local diffusion barriers, such as gaps within the bands forming radial alleys free of precipitate, transition from bands to speckled patterns and links between the bands called *anastomoses* [26]. Furthermore, a transient homogeneous colloid phase is observed before the formation of rings [27].

The classical analogy between Baló's concentric sclerosis and Liesegang rings relies on the supersaturation theory. An unknown myelinotoxic molecule is supposed to diffuse from the center of the lesion and induce demyelination after periodic precipitation. More precisely, Hallervorden et al. [2] suppose that the diffusing myelinotoxic substance induces a local formation of antibodies which inhibit myelin destruction. Protected regions, in which the toxin reacts with the antibodies, then behave as the precipitates in the Ostwald supersaturation theory by attracting the protective antibodies from the surrounding brain tissue. Demyelination thus occurs in the surrounding regions when they are reached by the toxin front, as they do not contain enough antibodies to remain protected. In this scenario, the preserved areas in the lesions correspond to the precipitation bands in Liesegang rings.

**Figure 2:**
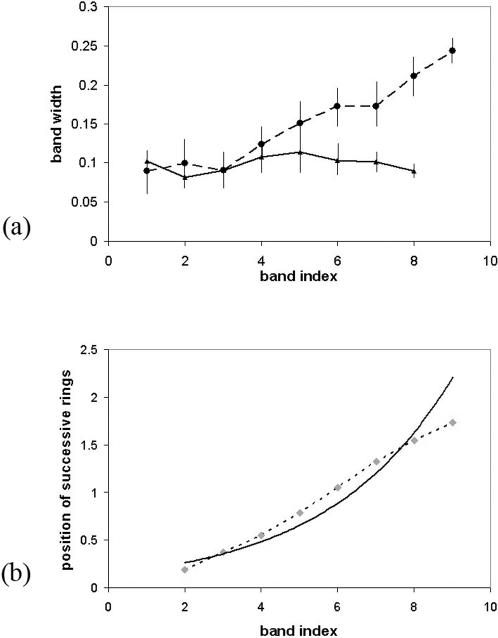
Space laws from 12 measures made on the cases of Baló (1928) Arch Neurol Psychiatr 19:242–264 and Hallervorden et al. [2]. (a) Damaged (dashed line) and preserved (solid line) myelin. (b) Averaged successive positions of destroyed myelin bands (dashed), compared to the classical exponential space law for the Liesegang rings (solid line) obtained from a logarithmic regression; y-axis label in *cm*s. The space law for concentric sclerosis is linear (correlation coefficient *r* ^2^ = 0,996). In all our mathematical models, the space law strongly depends on the shape of the activating front. As the biological nature of this front is unknown, selection between different scenarios (e.g. preconditioning model vs. local macrophage recruitment model) cannot be based on the space law. However the linear law for concentric sclerosis tends to indicate that an outer diffusing signal is unlikely.

The hypothesis of Hallervorden et al. [2] is a free interpretation of the supersaturation theory and has no biological basis. Furthermore, some Baló lesions display irregularities that bear some resemblance with the morphological characteristics of Liesegang rings that are not well explained by the supersaturation theory, such as branching patterns – close to the Liesegang anastomoses (figure 1a, 1d) – and speckled patterns – resembling the fragmentation of Liesegang rings when diffusion barriers occur [28]. Baló's concentric lesions become bands in the brainstem and the medulla, where diffusion zones are narrower than in the brain [29]. The initial non-periodic diffusion anomalies described in Baló's sclerosis [19] are also in line with the homogeneous colloid field found in the early phases of Liesegang ring formation. Finally, Baló's lesions do not respect any of the generic laws that characterize Liesegang rings (figure 2). The analogy between Baló's concentric sclerosis and Liesegang rings thus requires a revision (see table 1).

**Table 1:**
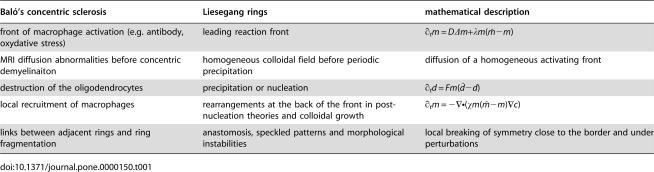
Analogies between Baló's sclerosis, Liesegang rings and the chemotactic description (model (3)-(4)-(5)).

Baló's concentric sclerosis	Liesegang rings	mathematical description
front of macrophage activation (e.g. antibody, oxydative stress)	leading reaction front	∂*_t_m* = *D*Δ*m*+λ*m*(*m̄* −*m*)
MRI diffusion abnormalities before concentric demyelinaiton	homogeneous colloidal field before periodic precipitation	diffusion of a homogeneous activating front
destruction of the oligodendrocytes	precipitation or nucleation	∂*_t_d* = *Fm*(*d̄* −*d*)
local recruitment of macrophages	rearrangements at the back of the front in post-nucleation theories and colloidal growth	∂*_t_m* = −∇•(χ*m*(*m̄* −*m*)∇*c*)
links between adjacent rings and ring fragmentation	anastomosis, speckled patterns and morphological instabilities	local breaking of symmetry close to the border and under perturbations

**Table 2:**
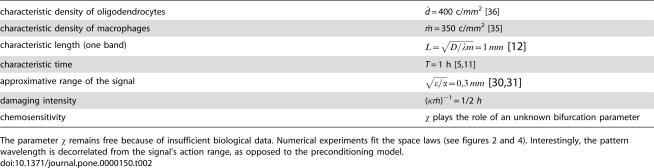
Numerical data on concentric demyelination used as mean values for the reduced parameters in numerical simulations.

characteristic density of oligodendrocytes	*d̄* = 400 c/*mm* ^2^ [36]
characteristic density of macrophages	*m̄* = 350 c/*mm* ^2^ [35]
characteristic length (one band)	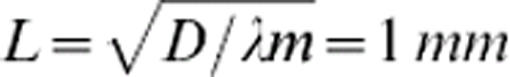 [12]
characteristic time	*T* = 1 h [5], [11]
approximative range of the signal	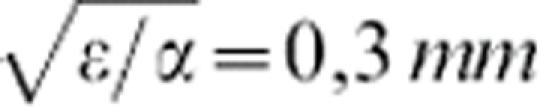 [30], [31]
damaging intensity	(κ*m̄* )^−1^ = 1/2 *h*
chemosensitivity	χ plays the role of an unknown bifurcation parameter

The parameter χ remains free because of insufficient biological data. Numerical experiments fit the space laws (see figures 2 and 4). Interestingly, the pattern wavelength is decorrelated from the signal's action range, as opposed to the preconditioning model.

## Analysis

### Mathematical reconsideration of the preconditioning theory

**Figure 3:**
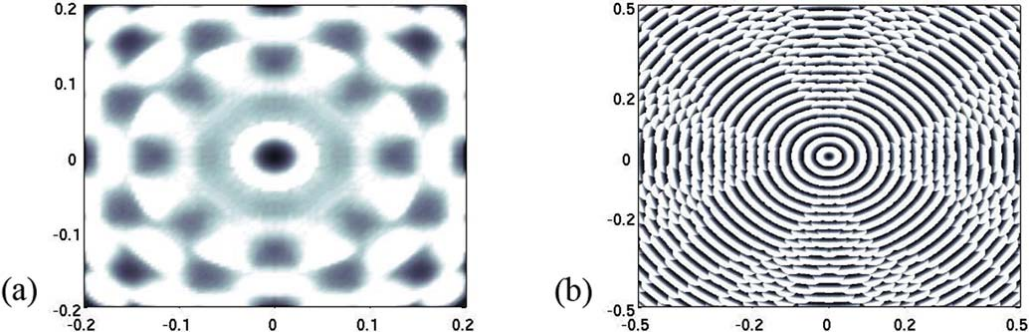
Apparition of concentric patterns under the preconditioning model (1)-(2). The protection factor is given by Heaviside function *P*(φ) = (*q*−φ)_+_ with a given threshold *q*. (a) We have replaced the preconditioning equation (2) with φ = K*d, the kernel *K* being a stiff Hill function with a 
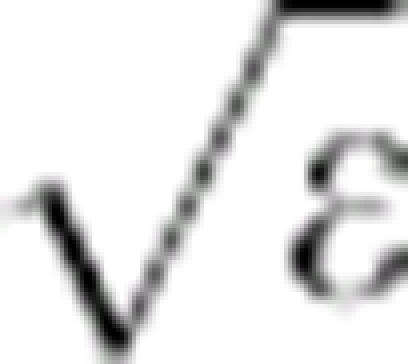
 range of action, 
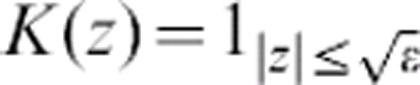
. Other parameters are ε = 0,4 and *q* = 0,1. Interestingly the range of action of the potential φ is larger than in figure 5 whereas the size of the domain is considerably smaller. In fact, this range fully determines the width of the bands. (b) Same illustration with a degenerated potential 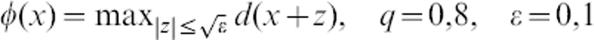. Destroyed oligodendrocytes are figured in black. The size of the domain is four times smaller than in figure 4 (axis labels in *cm*s), whereas the action range is similar.

**Figure 4:**
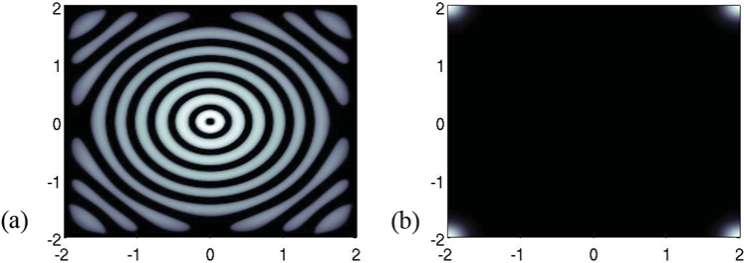
Transition between concentric patterns and plaques is driven by the structural parameter χ. Reduced parameters are ε̃ = 0,2, κ/λ = 4, χ̃ = 25 (a) and χ̃ = 8 (b). The pattern diameter is approximatively 4 cm (axis labels in *cm*s), and the final time is 24 h. These results fit the biological data (see table 2). Destroyed oligodendrocytes are figured in black. The white corners in (b) are boundary artifacts.

The preconditioning theory makes the hypothesis that a protective substance, secreted by the attacked oligodendrocytes, prevents demyelination at the borders of the radially expanding lesion. From the scenario sketched by Stadelmann et al. [18], we extract the following continuous model for (1) the destroyed oligodendrocytes and (2) the preconditioning potential.
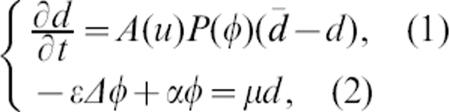
the outer variable *u* being an auto-immune damaging front and *A* (activation) and *P* (protection) being typical Heaviside functions associated with given thresholds. Subsequently ∂*_t_d* is zero unless *u* and φ are respectively above and below given thresholds. The elliptic equation (2) results from a quasi steady state approximation of the classical reaction–diffusion equation δ∂*_t_*φ = εΔφ+μ*d*−αφ. Brownian diffusion is not the only possibility (see figure 3). As suggested by the authors, this model also exhibits concentric ring formation. However, numerical analysis indicates that the range of the potential φ – a very strong hypothesis in the preconditioning theory – determines entirely the width of the protected areas. This leads to the confusing hypothesis of a long-range action for φ (about 100 cells of amplitude) which is not in accordance with the reported diffusion lengths of signaling molecules. In frog embryos, *TGFβ1* freely diffuses in a range of seven cell diameters (about 150 to 200 µm) and *activin* attains a diffusion distance of approximatively twelve cell diameters (350 µm) [30], [31]. Only the diffusion of small inorganic molecules can attain much larger ranges [32]. It thus seems improbable that the molecules involved in ischaemic preconditioning attain diffusion ranges more than ten times larger than the ones attained by key developmental factors. On the other hand, in our model (3)-(4)-(5) the width of preserved areas is characterized by a non-linear process driven by chemotaxis and closely matches the quantitative aspects observed in human lesions (compare figures 3 and 4).

Furthermore, it is particularly difficult to render numerically the apparition of rings using the preconditioning model (1)–(2), which only leads to the progression of a balanced moving boundary between the zero and the homogeneous states of myelin destruction. The key mechanism that may be lacking in preconditioning is the presence of two thresholds, as in Ostwald's scenario for Liesegang rings. As a matter of fact, in Ostwald's model, precipitation initiates when [*AB*]>q* and persists because the second threshold *q* is strictly lower than q*.

For periodic precipitation to occur in the preconditioning model 2, it would be nevertheless possible to increase the efficiency of the protection potential by using for instance φ = K*d, where the convolution kernel *K* has a plateau-shape (figure 3). In this case, however, φ has no clear biological significance.

Another situation where preconditioning could lead to banding would involve temporal discontinuities in front progression during the period necessary for the formation of a lesion. Nevertheless, successive multiple sclerosis attacks are never clinically observed in such a short period of time.

### Concentric demyelination: a chemotactic approach

The so-called post-nucleation theories of Liesegang ring formation – Ostwald's supersaturation being a pre-nucleation theory – are based on the hypothesis that a diffusing intermediate colloidal compound *C* interacts with *B* and aggregates to form the precipitate *D*:
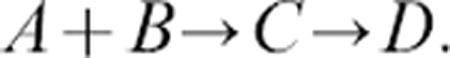
Post-nucleation theories consider that the first step of ring formation is the appearance of a homogeneous colloidal field subjected subsequently to competitive growth or coarsening between the colloidal particles *C* [33]. These theories allow a more accurate description of the diversity of Liesegang ring patterns [25], [34] and take into account the existence of the initial transient homogeneous colloidal state. The following scenario (table 1) for two-dimensional ring formation in Baló's sclerosis is based on the analogy with post-nucleation Liesegang ring formation theories. Namely it considers secondary rearrangement processes at the back of the structural front. It requires no hypothesis on concentric demyelination and makes very few assumptions about the molecular processes involved in the pathogeny of MS. Inactive macrophages are initially spread in the white matter. Their density is about *m̄*  = 350 c/mm^2^ [35]. An activation front travels in the white matter from a lesional center, which can be a blood vessel, and drives the macrophages into an auto-immune active state. This front can be an activating molecule like an antibody, or a wave of oxydative stress [10]. The activated macrophages attack the oligodendrocytes, which are evenly distributed at a density of *d̄*  = 400 c/mm^2^ [36]. Moreover, damaged cells and phagocyting macrophages produce a signal that attracts surrounding activated macrophages. This chemoattractant can be a pro-inflammatory cytokine, such as *TNF*α, *IL-1* and *IFNγ* [37]. Concentric lesions can develop in the first hours following a MS attack [11], and the MRI of patients one week after the clinical onset of the disease shows fully concentric lesions [5]. This indicates that a characteristic time scale of the order of one hour is relevant in the modelling (see table 2 for the whole set of parameters). These observations lead us to describe the dynamics of (3) the density *m* of activated macrophages, (4) the concentration *c* of the attraction signal and (5) the density *d* of destroyed oligodendrocytes.
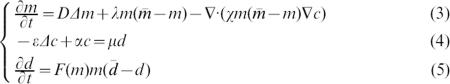
with *m̄*, *d̄* being characteristic densities of macrophages and oligodendrocytes respectively, involved into saturation phenomena in the activation and the recruitment of the macrophages (3) and in the destruction of the oligodendrocytes (5).

The simulations are initiated by a small, centered, bump of activated macrophages (*m*>0) issuing from a blood vessel. Boundary conditions are zero-flux for both *m* and *c* and have no influence on the formation of the structure before the front reaches the limits of the domain. The cell flux in (3) decomposes into an activation front, a small diffusion contribution (∂*_t_m*−δΔ*m*) and a drift enhanced by the gradient of the diffusing signal (∂*_t_m*+∇•(χ(*m*)*m*∇*c*)). The elliptic equation (4) is the result of a quasi steady state approximation. The front propagation in (3) follows a Fisher-type equation, but the final pattern is independent of this particular choice. The front can in fact be a diffusing molecule which does not interact with white matter cells, or a molecule involved in more complex interactions with macrophages, like an antibody. Both cases lead to the same behavior, except for the spacing laws: a traveling front with constant speed induces a linear position increase, whereas a diffusing front generates quadratic increasing (see also figure 2). Baló's sclerosis seems to follow a linear spacing law and may thus not be accurately described by a mechanism involving diffusion alone. The damaging function *F* can be chosen almost arbitrarily as long as it is both positive and increasing. We set *F*(*m*) = κ*m*/(*m̄*+*m*).

Chemotaxis is the collective motion of cells induced by a chemical gradient. Aggregation may occur when the cells themselves produce attractive substances, creating a non-linear coupling and leading to a blow-up of the cell density. We have opted for the well-studied Patlak, Keller & Segel (PKS) model [38], [39]. If *M*, *D*, χ denote respectively the total number of cells, the diffusion of cells and the chemosensitivity in this system, the reduced structural parameter χ*M*/*D* controls bifurcation and determines the appearance of bundles, as was first conjectured [40], then proved rigorously [41], [42]. Recent advances have focused on the precise threshold value between spread of cells and formation of clusters [43], [44] and on the qualitative description of the system including nonlinear coefficients [45]. The volume-filling approach [46], [47], which can be viewed as an extreme saturation effect, plays a major role in our model (in equation (3)).

We set the reduced variables and parameters as follows:
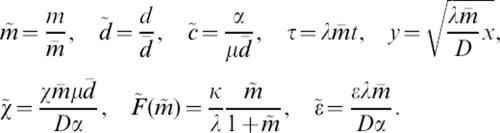
In particular, the ratio *r* = κ/λ balances the speed of the front and the intensity of the macrophages in damaging the myelin.

We obtain the following non-dimensionalized system,
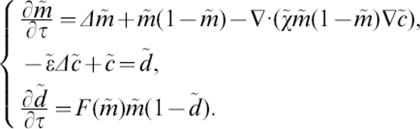
6Only three parameters remain, namely the reduced chemosensitivity χ̃, the reduced chemical diffusivity ε̃ and the ratio *r* = κ/λ.

According to the PKS model, structure formation in (3)-(4)-(5) is driven by the reduced parameter χ̃ = χ*m̄*μ*d̄*/*D*α: a bifurcation occurs between a plaque state – for small values of χ̃ – and a concentric pattern – for larger values of χ̃ (figure 4).

**Figure 5:**
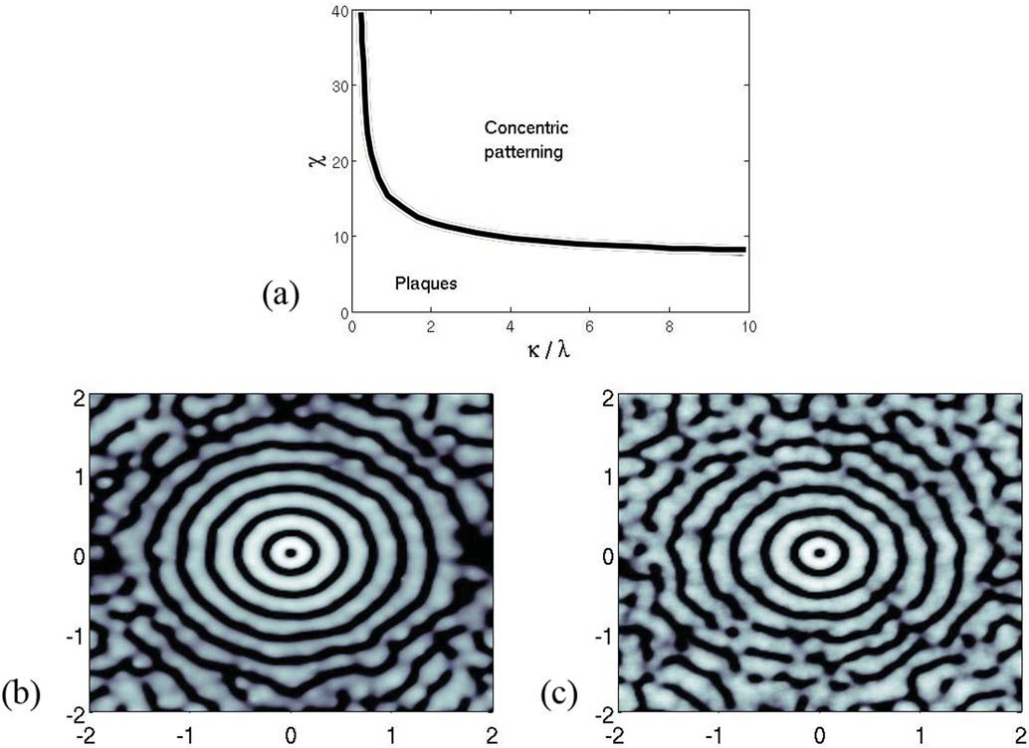
(a) Bifurcation diagram for fixed reduced parameter ε̃ = 0,1. Only two alternative patterns arise: concentric rings or plaques apparition. (b) Imposed white noise perturbation with relative standard deviation σ = 0,2 to the chemical diffusion coefficient ε̃ = 0,2. Other parameters are χ̃ = 25 and κ/λ = 5. (c) White noise perturbation with relative standard deviation σ = 0,1 to the damaging factor κ/λ = 5. We set ε̃ = 0,2 and χ̃ = 25. Destroyed oligodendrocytes are figured in black. The damaging factor is more sensible to perturbation that the chemical diffusion coefficient; increasing the standard deviation breaks the symmetry of the pattern, except around the origin. Anastomoses are noted in (c) far from the regions affected by the boundary effects.

High values of χ̃ reflect the aggressivity of the demyelinating process and are obtained when *m̄* is large. In fact, the destruction of oligodendrocytes is correlated with the number of macrophages [36]. Interestingly, when there is a major oligodendroglial destruction (that is when the reduced parameter *r* = κ/λ is large), patterning is favored (see figure 5a). The fact that the oligodendroglial destruction rate in Baló's sclerosis is generally larger than in classical forms of multiple sclerosis [9] is in line with the structurating role of cellular aggressivity.

Highly robust concentric patterning is supported by numerical evidence. The model only produces heterogeneous concentric damaged areas and homogeneous demyelinated plaques (figure 5a). Pattern formation does not depend on initial conditions: an asymmetric source of activated macrophages leads to a perfectly round-shaped pattern (data not shown). Furthermore, concentric symmetry is very well conserved by different types of random perturbation. White noise perturbation of chemical diffusion and randomization of the damaging function conserve the rings and induce the appearance of patterns close to the anastomoses and the peripheral fragmentation of the real lesions (see figures 5b, 5c). The concentric pattern is maintained as long as the destroyed oligodendrocytes produce the attractive potential *c*. This is justified because the characteristic time of our model is shorter than the relaxation time of the macrophages. The secondary dispersion of macrophages when oligodendrocytes do not produce *c* anymore could explain that concentric lesions disappear in some MRI follow-up cases [4].

### Conclusion

The non-linear approach involving a chemotactic mechanism for the lesional process allows a realistic description of Baló's sclerosis lesions without making any assumptions about specific cellular process. The variation range of the parameter χ̃ shows a transition from a homogeneous plaque state characteristic of the classical MS lesions to a concentric, Baló-type, pattern. As our set of hypotheses only involves cellular events common to most subtypes of MS, our results are compatible with the hypothesis that Baló's sclerosis is a borderline form of MS. The high prevalence of this disease in South-Eastern Asian countries [5], [7], [29] may then be the result of extreme behaviors – for instance when the reduced chemotactic parameter χ̃ is beyond the bifurcation threshold, and may reflect the crucial role of cellular aggressivity in pattern formation.
